# Sulfation Pathways During Neurodevelopment

**DOI:** 10.3389/fmolb.2022.866196

**Published:** 2022-04-14

**Authors:** Taylor Clarke, Francesca E. Fernandez, Paul A. Dawson

**Affiliations:** ^1^ School of Behavioural and Health Sciences, Faculty of Health Sciences, Australian Catholic University, Banyo, QLD, Australia; ^2^ Mater Research Institute, University of Queensland, Brisbane, QLD, Australia

**Keywords:** sulfate, brain, embryological, fetal, gene expression, neurological dysfunction

## Abstract

Sulfate is an important nutrient that modulates a diverse range of molecular and cellular functions in mammalian physiology. Over the past 2 decades, animal studies have linked numerous sulfate maintenance genes with neurological phenotypes, including seizures, impaired neurodevelopment, and behavioral abnormalities. Despite sulfation pathways being highly conserved between humans and animals, less than one third of all known sulfate maintenance genes are clinically reportable. In this review, we curated the temporal and spatial expression of 91 sulfate maintenance genes in human fetal brain from 4 to 17 weeks post conception using the online Human Developmental Biology Resource Expression. In addition, we performed a systematic search of PubMed and Embase, identifying those sulfate maintenance genes linked to atypical neurological phenotypes in humans and animals. Those findings, together with a search of the Online Mendelian Inheritance in Man database, identified a total of 18 candidate neurological dysfunction genes that are not yet considered in clinical settings. Collectively, this article provides an overview of sulfate biology genes to inform future investigations of perturbed sulfate homeostasis associated with neurological conditions.

## Introduction

Sulfate is essential for healthy growth and development (Dawson, 2011). Supplied from the diet and catabolism of sulfur-containing amino acids, intracellular sulfate is transformed into 3′-phosphoadenosine 5′-phosphosulfate (PAPS) which is the universal sulfate donor for sulfate conjugation (sulfonation) to a wide range of endogenous and exogenous molecules via sulfotransferases (Mulder and Jakoby, 1990). Sulfonation biotransforms the physiological properties of molecules, including: (i) inactivation of steroids, thyroid hormone and neurotransmitters (Darras et al., 1999; Richard et al., 2001; Dawson, 2012); (ii) detoxification of xenobiotics and certain pharmaceutical drugs (McCarver and Hines, 2002); and (iii) maintenance of tissue structure and function via the sulfate content of glycosaminoglycans (Yamaguchi, 2001). Sulfatases remove sulfate from substrates to maintain the required balance of sulfonated to unconjugated substrates (Hanson et al., 2004). Disturbances in any of these sulfation pathways has the potential for adverse physiological consequences.

In recent years, interest in human sulfate biology has expanded following animal studies that show adverse phenotypes linked to sulfate biology genes (Langford et al., 2017). Neurological dysfunction is one of the most predominant phenotypes linked to disturbances of sulfate biology. This is not surprising when considering that sulfonation biotransforms numerous target molecules in the brain, including inactivation of thyroid hormone, metabolism of neurotransmitters (e.g., serotonin, noradrenaline and dopamine) and modulation of neurosteroid actions on GABA_A_, *N*-methyl-D-aspartate (NMDA) glutaminergic and ơ-opioid receptors (Rivett et al., 1982; Kríz et al., 2008). Sulfonation of proteoglycans (e.g., heparan sulfate) and cerebroside sulfate also plays an important role in maintaining the structure and function of brain tissue (Schwartz and Domowicz, 2018). Animal studies have also shown that hyposulfataemia leads to reduced sulfonation capacity and abnormal behavioral phenotypes, including impaired memory, increased anxiety and seizures in mice (Dawson et al., 2003; Dawson et al., 2004; Dawson et al., 2005). More recent studies have shown that reduced transfer of sulfate across the blood-brain barrier of neonatal mice leads to: (i) decreased heparan sulfate levels in the subventricular zone (SVZ) and rostral migratory stream; (ii) impairment in perineuronal net formation (which contains sulfonated proteoglycans) in the hippocampus and somatosensory cortex; (iii) increased neural stem cell proliferation and decreased neuronal maturation in the SVZ; (iv) seizures; and (v) impaired behavioral phenotypes, including impaired long-term spatial memory and defects in social interaction and social memory (Zhang et al., 2020). Despite the diverse roles for sulfate during neurodevelopment, most human sulfate biology genes have not yet been considered in clinical genetics.

To date, 91 genes are known to contribute to maintaining sulfate homeostasis, including those encoding: sulfate transporters; PAPS synthetases and transporters; enzymes in the pathway of sulfate generation; cytosolic and membrane-bound sulfotransferases; and sulfatases (Langford et al., 2017). However, only 24 of these genes are currently considered in clinical genetics, with 16 of those genes linked to neurological dysfunction (Table 1). Animal studies have shown additional sulfate biology genes are linked to adverse neurological phenotypes (Langford et al., 2017), suggesting that the number of clinically reportable genes is currently underestimated. This warrants further investigation into the role of sulfate biology genes in human neurophysiology.

**TABLE 1:** Sulfate biology genes that are either clinically reportable or not yet captured in clinical genetics.

^a^Clinically reportable genes			Genes that have been investigated in research settings but are not yet captured in clinical resources
Green	Amber	Red	
**Sulfate transporters**			
*SLC4A1*	*SLC26A1*		*SLC13A1, SLC13A4, SLC26A6, SLC26A9, SLC26A11*
*SLC26A2*			
*SLC26A3*			
*SLC26A7*			
*SLC26A8*			
**PAPS synthetases**			
*PAPSS2*			*PAPSS1*
**PAPS transporters**			
			*SLC35B2, SLC35B3*
**Key enzymes in the pathways of sulfate generation**			
^b^ *CBS*	^b^ *CTH*		*CDO1, SQOR, TST*
*GOT1*			
^b^ *SUOX*			
**Cytosolic sulfotransferases**			
*SULT2B1*			*SULT1A1, SULT1A2, SULT1A3, SULT1A4, SULT1B1*
			*SULT1C2, SULT1C3, SULT1C4, SULT1E1, SULT2A1*
			*SULT4A1, SULT6B1*
**Membrane-bound sulfotransferases**			
^b^ *CHST3*	*CHST11*	^b^ *CHST8*	*CHST2, CHST4, CHST5, CHST7, CHST9*
^b^ *CHST6*	^b^ *HS2ST1*		*HS3ST6, CHST10, CHST12, CHST13, CHST15, GAL3ST1*
^b^ *CHST14*		*^b^* *HS6ST1*	*GAL3ST2, GAL3ST3, GAL3ST4, HS3ST1, HS3ST2*
^b^ *NDST1*			*HS3ST3A1, HS3ST3B1, HS3ST4, HS3ST5, HS6ST2, HS6ST3, NDST2, NDST3, NDST4, TPST1, TPST2, UST*
**Sulfatases and sulfatase modifying factors**			
^b^ *ARSA*			*ARSD, ARSF, ARSG, ARSH, ARSJ, ARSK, SULF1, SULF2*
^b^ *ARSB*			*SUMF2*
^b^ *STS*			
^b^ *ARSE*			
^b^ *ARSI*			
^b^ *GALNS*			
^b^ *GNS*			
^b^ *IDS*			
^b^ *SGSH*			

a Genes captured in clinical resources and coded green (high level of evidence), amber (medium level evidence, not yet used) and red (no strong evidence, do no use) by searching the online PanelAPP (https://panelapp.agha.umccr.org/) consensus diagnostic gene panels (Martin et al., 2019) 18–19 January 2022.

b Linked to adverse neurological phenotype.

In this study, we curated the temporal and spatial mRNA expression patterns of all known sulfate biology genes in the developing human fetal brain between 4 and 17 weeks gestation, corresponding to a critical time for nervous system formation *in utero*. We show moderate to abundant mRNA expression levels of 62 genes, suggesting a critical role of sulfate in early neurodevelopment. Additionally, we collated 18 sulfate biology genes implicated in atypical neurological function from the animal and human research literature which are currently not captured in clinical resources. Overall, these findings provide data for future investigations of sulfate biology genes associated with neurological dysfunction.

## Sulfate Biology Gene Expression in Human Fetal Brain

**FIGURE 1:**
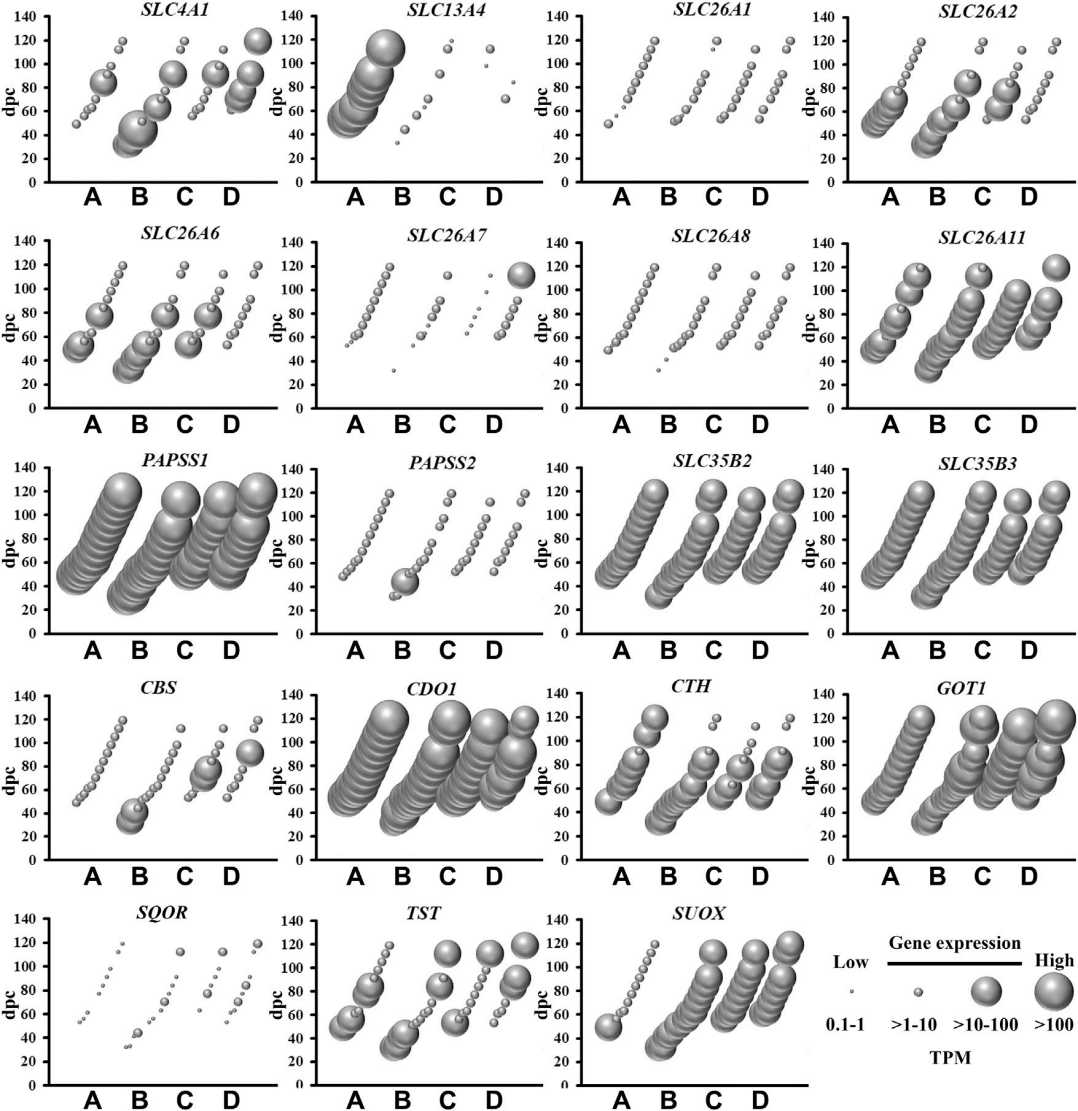
Spatial and temporal mRNA expression of sulfate transporters, PAPS synthetases and transporters, and enzymes in the pathways of sulfate generation in fetal brain regions **(A)** cerebrum, **(B)** brainstem, **(C)** diencephalon, and **(D)** cerebellum. dpc, days post-conception. TPM, transcripts per million.

**FIGURE 2:**
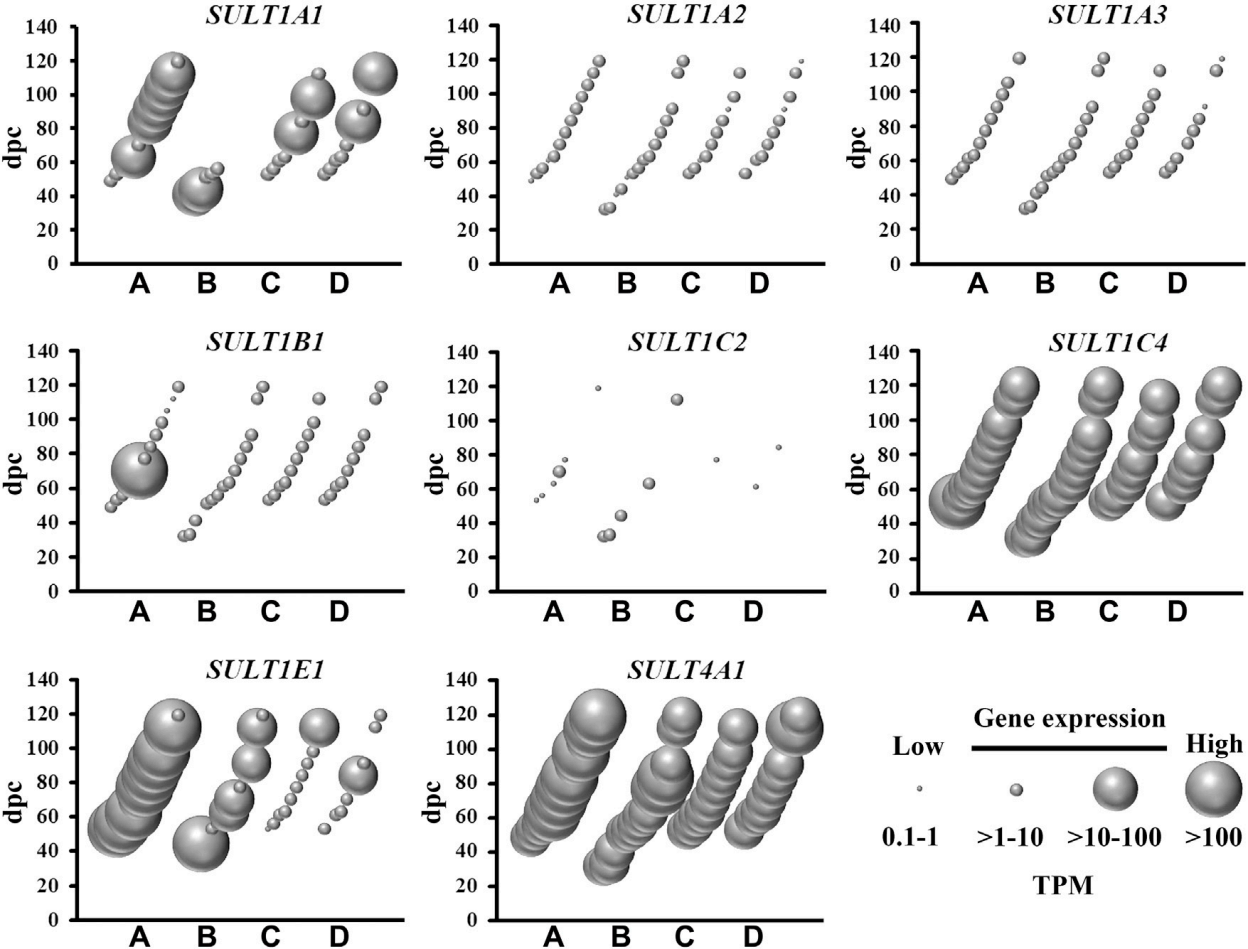
Spatial and temporal mRNA expression of cytosolic sulfotransferases in fetal brain regions **(A)** cerebrum, **(B)** brainstem, **(C)** diencephalon, and **(D)** cerebellum. dpc, days post-conception. TPM, transcripts per million.

**FIGURE 3:**
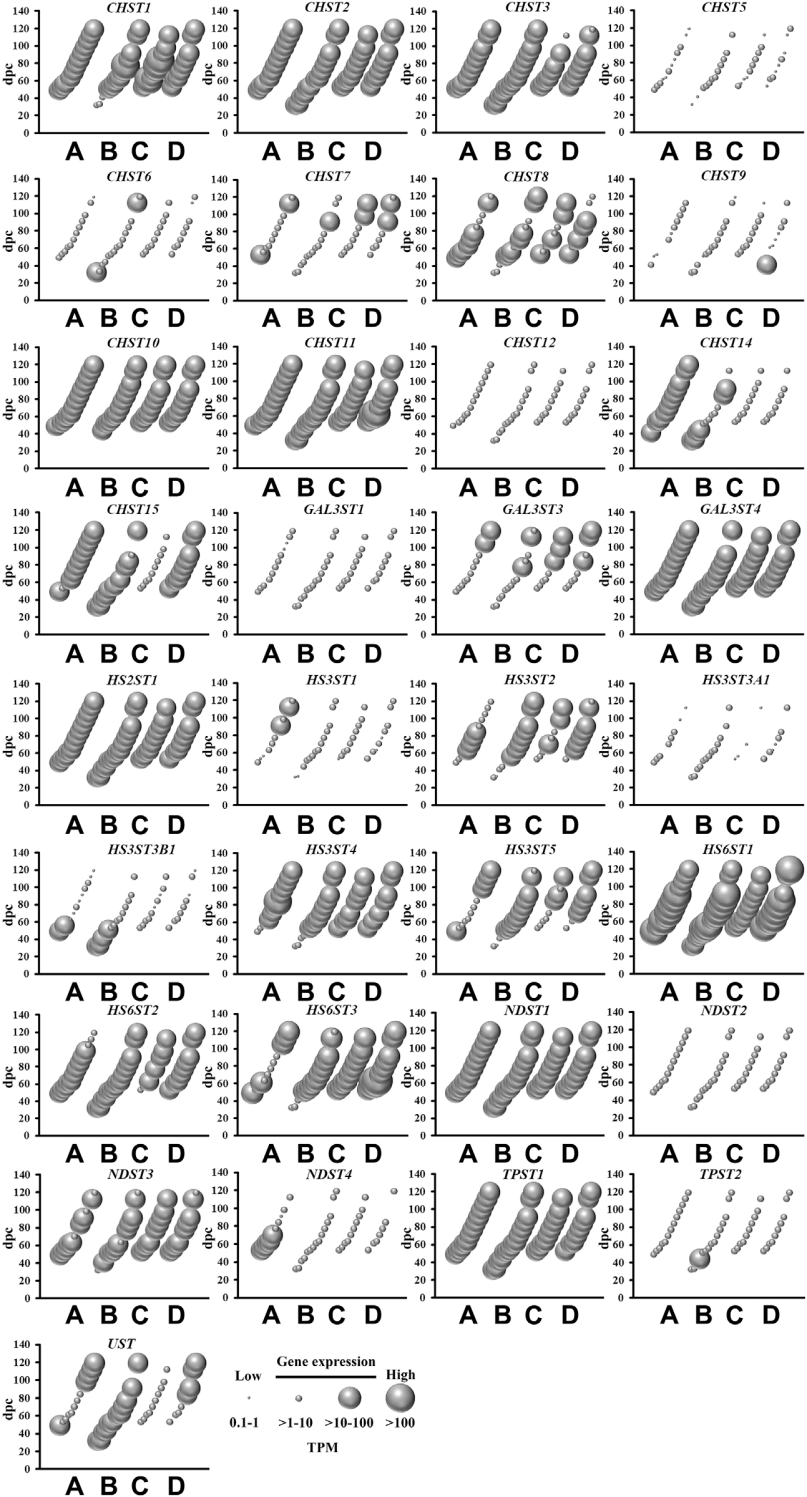
Spatial and temporal mRNA expression of membrane-bound sulfotransferases in fetal brain regions **(A)** cerebrum, **(B)** brainstem, **(C)** diencephalon, and **(D)** cerebellum. dpc, days post-conception. TPM, transcripts per million.

**FIGURE 4:**
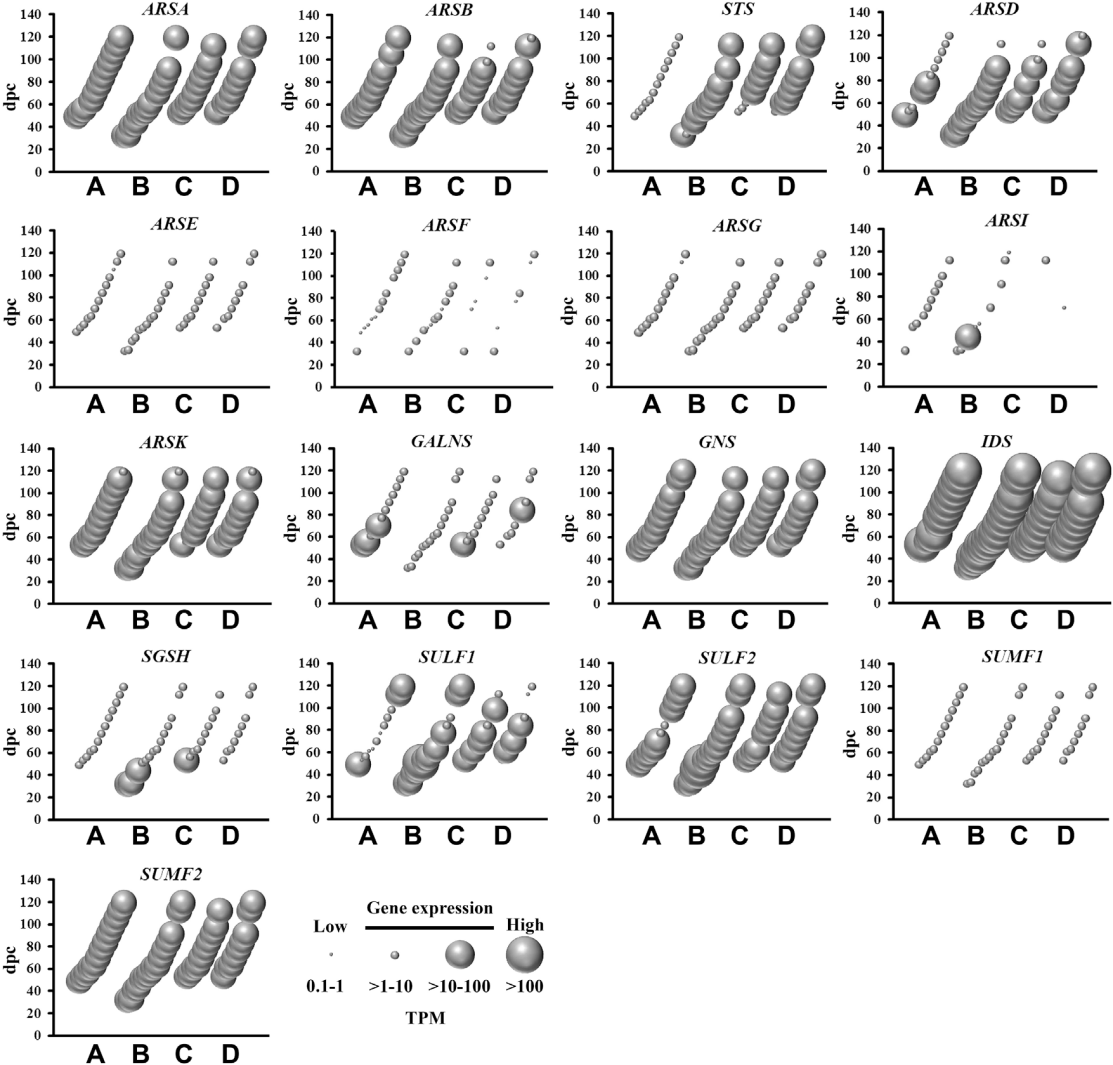
Spatial and temporal mRNA expression of sulfatases and sulfatase modifying factors in fetal brain regions **(A)** cerebrum, **(B)** brainstem, **(C)** diencephalon, and **(D)** cerebellum. dpc, days post-conception. TPM, transcripts per million.

We obtained data of gene expression in the human fetal brain from the online Human Developmental Biology Resource (HDBR) Expression (Lindsay et al., 2016) (https://www.hdbr.org/) from 6 November 2021 to 12 January 2022. The HDBR is one of the rare human fetal tissue databases providing gene expression under 8 weeks post-conception compared to other databases (Johnson et al., 2009; Sunkin et al., 2013), which is essential for getting a better understanding of brain development since neurulation starts after 3 weeks post conception (O'Rahilly and Müller, 1994). Taking in account that brain development is dynamic, HDBR reports anatomical regionalisation gene expression results, which is also essential for mapping the differential pattern of gene expression related to selected brain regions compared to using full brain tissues (Ono et al., 2017). Data were available for four brain regions (cerebrum, brainstem, diencephalon and cerebellum) from 4 to 17 weeks post-conception. The gene expression levels for each tissue sample were averaged for each set of technical replicates, and then quantile normalized within each set of biological replicates using limma. Finally, they are averaged for all biological replicates. Specifically, we used transcripts per million (TPM) mapped reads-normalized RNA-Seq data to plot results for each gene. Of the 91 sulfate biology genes, 16 were abundantly expressed (>100 TPM) in at least one brain region at one gestational time point, 46 genes had moderate (>10 to 100 TPM) mRNA expression, and 15 genes had lower (≥0.1 to 10 TPM) mRNA expression levels (Figures 1–4). Fourteen genes (*SLC13A1*, *SLC26A3*, *SLC26A9*, *SULT1A4*, *SULT1C3*, *SULT2A1*, *SULT2B1*, *SULT6B1*, *CHST4*, *CHST13*, *GAL3ST2*, *HS3ST6*, *ARSH* and *ARSJ*) had undetectable or negligible (<0.1 TPM) mRNA expression levels (data not shown). The limitations of these data are that mRNA levels may: (i) not be representative of encoded protein level; (ii) not be predictive of functional impact when abnormal, for example altered expression of highly-expressed genes does not necessarily result in a more severe neurodevelopmental phenotype than altered expression of lowly-expressed genes; (iii) be redundant as other family member genes may compensate for altered expression level of a particular gene; (iv) not identify any sex-specific effects as the data are derived from pooled samples, which warrants future investigations to compare sulfate biology gene expression between male and female samples. Furthermore, our focus on genes expressed in the brain doesn’t take into account any of the sulfate biology genes expressed in other tissues that may influence neurodevelopment, such as the thyroid or adrenal gland or testis that could theoretically influence neurodevelopment through endocrine effects. Nonetheless, the temporal and spatial mRNA expression profiles of sulfate biology genes reported in the present study provide information for the next phase of research into understanding the regulation of sulfate homeostasis in the developing brain.

### Genes Involved in Sulfate and PAPS Supply

Amongst the most abundantly expressed sulfate biology genes at all gestational time points are those involved in: (i) transporting sulfate from circulation to brain across the blood brain barrier (*SLC13A4*) (Zhang et al., 2020); (ii) the major (*CDO1*) and minor (*CTH*) biochemical pathways of sulfate generation (Dawson et al., 2020); and (iii) synthesis (*PAPSS1*) and transportation (*SLC35B2* and *SLC35B3*) of PAPS (Langford et al., 2017) (Figure 1). *SLC13A4* mRNA expression was specifically expressed in the cerebrum, indicating that sulfate transport from circulation likely contributes an important role in sulfate supply in early neurodevelopment. Of great interest are the data showing abundant mRNA expression of those genes involved in pathways of sulfate generation from the sulfur-containing amino acid cysteine. Previous studies suggested that the developing fetus has negligible capacity to generate sulfate, based on low *CDO1* and *CTH* mRNA expression levels in the liver (Gaull et al., 1972; Loriette and Chatagner, 1978). However, a more recent animal study showed the expression of *Cdo1* mRNA in the developing mouse fetus from mid-gestation (Rakoczy et al., 2015). The present study shows abundant expression of *CDO1*, suggesting that the fetal brain most likely generates sulfate, in addition to obtaining sulfate from circulation via *SLC13A4*. The present study also shows that *PAPSS1* is the most abundantly expressed sulfate biology gene in all four selected regions of the fetal brain, suggesting that this gene encodes for the predominant PAPS synthetase in fetal brain tissue, rather than *PAPSS2* which is expressed in cartilage and linked to developmental dwarfism disorders (Bownass et al., 2019). In addition, the findings of abundant *SLC35B2* and *SLC35B3* mRNA expression suggest that PAPS is actively transported in the golgi of fetal brain cells for sulfonation reactions via membrane-bound sulfotransferases. Collectively, these findings of abundant mRNA expression of genes involved in sulfate supply and generation, as well as generation and transport of PAPS, suggest that sulfate has an important physiological role in early fetal neurodevelopment.

### Sulfotransferase Genes

There are two classes of sulfotransferases based on their protein sub-cellular localization: (i) cytosolic sulfotransferases which sulfonate neurotransmitters, bile acids, xenobiotics and steroids; and (ii) membrane-bound sulfotransferases that sulfonate proteoglycan and lipid molecules (Gamage et al., 2006). The present study shows abundant mRNA levels of four cytosolic (Figure 2) and 22 membrane-bound (Figure 3) sulfotransferases. *SULT4A1* is the most abundantly expressed cytosolic sulfotransferase mRNA in all four fetal brain regions. Whilst the substrate of SULT4A1-mediated sulfonation has not yet been identified, a recent study suggests it plays a role in the regulation of neuronal morphology and synaptic activity (Culotta et al., 2020). Abundant mRNA levels of *SULT1E1* and *SULT1C4*, suggest that these two sulfotransferases, that sulfonate estrogenic compounds (Dawson, 2012), may regulate hormonal signalling pathways during fetal neurodevelopment. Interestingly, *SULT1E1* mRNA is mainly expressed in cerebrum, which is a critical region in hormonal regulation due to its development into the thalamic and hypothalamic structures modulating endocrine body system. *SULT1A1* mRNA expression was abundant in the cerebrum, with lower levels detected in the other three brain regions. SULT1A1 sulfonates phenolic compounds, and while it has a major role of Phase II metabolism in the liver (Pacifici, 2005), it is also proposed to contribute to the detoxification and clearance of drugs and toxins in the brain (Salman et al., 2009). It is intriguing to see a single burst of abundant *SULT1B1*, iodothyronine sulfotransferase (Stanley et al., 2005), mRNA expression at 10 weeks post-conception. This time point coincides with the early stage of neuronal migration, a neurodevelopmental process that relies on the action of thyroid hormone which is regulated by SULT1B1-mediated sulfonation (Wang et al., 1998; Morreale de Escobar et al., 2004). In addition to the cytosolic sulfotransferases, the majority of all known membrane-bound sulfotransferases were abundantly expressed (Figure 3), including: carbohydrate sulfotransferases *CHST1*, *CHST2*, *CHST3*, *CHST8*, *CHST10*, *CHST11*, *CHST14,* and *CHST15*; galactose 3-O-sulfotransferases *GAL3ST3* and *GAL3ST4*; heparan sulfate 2-O-sulfotransferase *HS2ST1*; heparan sulfate 3-O-sulfotransferases *HS3ST2*, *HS3ST4,* and *HS3ST5*; heparan sulfate 6-O-sulfotransferases *HS6ST1*, *HS6ST2* and *HS6ST3*; heparan N-deacetylase/N-sulfotransferases *NDST1* and *NDST*; tyrosylprotein sulfotransferase *TPST1*; and uronyl 2-sulfotransferase *UST*. Overall, these findings suggest the sulfonation of numerous substrates in the developing fetal brain.

### Sulfatase Genes

The removal of sulfate from lipids, glycosaminoglycans and steroids is mediated by sulfatases (Hanson et al., 2004), of which nine are abundantly expressed in the fetal brain (Figure 4). This is relevant to sulfatases that target a range of substrates in the central nervous system, including sulfoglycolipids (ARSA), proteoglycans (ARSB, ARSK, GNS, IDS, SULF1 and SULF2) and steroids (STS). With the exception of *STS*, all of these sulfatase genes were expressed in all four brain regions over the 4–17 weeks gestational period. *STS* mRNA level is low in cerebrum when compared to brainstem, diencephalon and cerebellum, suggesting that steroid activity is spatially confined to the latter three brain regions during early neurodevelopment. *SULF1* and *SULF2* mRNA, highly expressed in developing fetal brain, lead to the formation of enzyme removing 6-O-sulfate groups of proteoglycans and modulating morphogen Shh gradient during embryonic development. By modifying the Shh gradient in the embryo, the regulation of motorneuron to oligodendrocyte precursor cell fate change is significantly affected in the developing ventral spinal cord (Jiang et al., 2017). The present study also shows abundant sulfatase modifying factor 2 (*SUMF2*) mRNA levels in comparison to low *SUMF1* mRNA levels, indicating that SUMF2 is the major sulfatase regulating factor in early fetal brain tissue. There were short bursts of increased *ARSI*, *GALNS* and *SGSH* mRNA expression that were spatially enriched, suggesting a localized and temporary requirement for these glycosaminoglycans metabolizing sulfatases. Taken together, the abundant mRNA expression of numerous sulfatases indicates an active process of regulating sulfate content in the developing fetal brain.

## Candidate Neurological Dysfunction Sulfate Biology Genes

Despite the mRNA expression of 62 sulfate biology genes in the fetal brain (Figures 1–4), only 12 of these genes are considered for neurological dysfunction in routine clinical genetics (Table 1). As an approach to inform clinical geneticists and potentially expand the list of clinically reportable genes, we undertook a systematic online search (10–23 September 2021). We used PubMed and Medline to identify sulfate biology genes reported to be associated with atypical neurological phenotypes in humans using the search terms: (“brain” or “cerebellum” or “cerebral” or “neurodevelop”) and (“gene” or “dna” or “gene expression regulation” or “gene expression” or “gene genetic regulation”) and (“sulphate” or “sulfate” or “SO4”). We included articles that associated any of the sulfate biology genes with neurological conditions. All references used in this study were peer-reviewed research articles published in English between January 2001 and September 2021. In addition, we used OMIM (https://www.ncbi.nlm.nih.gov/omim/?term=) to expand our search prior to 2001 and to include any research articles not captured in our literature search. Overall, our research literature and OMIM searches identified 42 research articles that report the involvement of sulfate biology genes in neurological dysfunction.

From the curated list of research articles, we identified 18 candidate neurological dysfunction genes that are not yet clinically reportable (Table 2). Ten of these genes were reported from animal studies (*SLC13A1*, *SLC13A4*, *SLC35B2*, *CDO1*, *CHST12*, *CHST15*, *NDST2*, *NDST3*, *SULF2* and *UST*), 2 from human and animal studies (*ARSG* and *SULF1*) and 6 from human research studies (*SQOR*, *TST*, *SULT4A1*, *CHST10*, *HS6ST2* and *ARSK*). Thirteen of these genes are abundantly expressed in the human brain between 4 and 17 weeks gestation, whereas *SQOR*, *CHST12*, *NDST2* and *ARSG* are expressed at low level (Figures 1–4) and *SLC13A1* mRNA level is negligible (data not shown). The *SLC13A1* gene is primarily expressed in the proximal tubule of the kidney where it mediates sulfate reabsorption (Ullrich and Murer, 1982; Lee et al., 2005). Disruption of human *SLC13A1* and mouse *Slc13a1* causes renal sulfate wasting and hyposulfataemia, which is proposed to reduce sulfate supply to the brain (Dawson et al., 2003; Bowling et al., 2012). Sulfide:quinone oxidoreductase (SQOR) is an enzyme in the minor pathway of sulfate generation (Dawson, 2013) and its loss leads to excess hydrogen sulfide levels that cause encephalopathy and Leigh disease (Friederich et al., 2020). Individuals with *SQOR* mutations first present with coma in early childhood (Friederich et al., 2020), suggesting a physiological role for *SQOR* in the brain beyond the 17-week gestational end point used to assess gene expression in the present study. Increasing *CHST12*, *NDST2* and *ARSG* mRNA levels in brain at developmental ages beyond 17 weeks gestation (Humphries et al., 1998; Hiraoka et al., 2000; Khateb et al., 2018), also explains their low mRNA levels presented in this study. Nonetheless, the 18 genes listed in Table 2, warrant further research to determine whether these candidate neurological dysfunction genes are to be considered in clinical genetics.

**TABLE 2:** Neurological phenotypes reported for sulfate biology genes that are not yet captured in clinical genetics.

Gene	Phenotypes (References)
**Sulfate transporters**	
*SLC13A1*	Mice: seizures, behavioural abnormalities (Dawson et al., 2003; Dawson et al., 2004; Dawson et al., 2005)
*SLC13A4*	Mice: impaired social behavior and neurogenesis (Zhang et al., 2019; Zhang et al., 2020)
**PAPS transporters**	
*SLC35B2*	*Drosophila*: pupal lethality (Kamiyama et al., 2003)
**Key enzymes in the pathways of sulfate generation**	
*CDO1*	Mice: fetal death and irregular shaped cranium (Ueki et al., 2011)
*SQOR*	Human: encephalopathy, seizures, coma, Leigh syndrome-like brain lesions (Friederich et al., 2020)
*TST*	Human: Leber optic atrophy (Cagianut et al., 1981)
**Cytosolic sulfotransferases**	
*SULT4A1*	Human: perturbed dendritic morphology and synaptic activity (Culotta et al., 2020)
**Membrane-bound sulfotransferases**	
*CHST10*	Human: T cell invasion and nerve damage during inflammation (Dasgupta et al., 2009)
*CHST12*	Mice: adverse cerebellar development (Ishii and Maeda, 2008)
*CHST15*	Mice: adverse cerebellar development (Ishii and Maeda, 2008)
*HS6ST2*	Human: Global developmental delay, intellectual disability, enlarged lateral ventricles (Paganini et al., 2019)
*NDST2*	Mice: Induction of neural cells (Forsberg et al., 2012)
*NDST3*	Mice: subtle behavioral abnormalities (Pallerla et al., 2008)
*UST*	Mice: adverse cerebellar development (Ishii and Maeda, 2008)
**Sulfatases and sulfatase modifying factors**	
*ARSG*	Human: Usher syndrome, Type IV (Abad-Morales et al., 2020; Fowler et al., 2021; Peter et al., 2021). Dog: cerebellar ataxia, neuronal ceroid lipofuscinosis (Abitbol et al., 2010)
*ARSK*	Human: Mucopolysaccharidosis type X (Verheyen et al., 2021)
*SULF1*	Human: microcephaly, diffuse or multifocal cerebral dysfunction (Day-Salvatore and McLean, 1998). Mice: Reduced hippocampal spine density and impaired long-term potentiation in CA1 (Kalus et al., 2009)
*SULF2*	Mice: Impaired generation of Olig2-expressing pMN-derived cell subtype (Ohayon et al., 2019). Mice: Congenital hydrocephalus and impaired spatial learning (Kalus et al., 2009)
*SULF1/2*	Mice: Corticospinal tract defects (Okada et al., 2017)

## Summary

In this study, we show the moderate to abundant mRNA expression of 62 sulfate biology genes in the human fetal brain between 4 and 17 weeks post-conception, a critical period in nervous system formation. This relatively large number of genes highlights the physiological relevance of sulfate in neurodevelopment and is a testament to the genetic complexity of maintaining sulfate homeostasis. However, sulfate biology is underappreciated in clinical settings, with only 12 genes routinely considered for certain neurological conditions. Our finding of an additional 18 genes reported in the research literature with neurological dysfunction, warrants future studies to validate the pathogenetics of these genes in human brain. Expanding the list of clinically reportable sulfate biology genes has the potential for improved genetic counselling, and potentially for developing therapeutic approaches towards clinical treatments. Overall, this study curated a list of sulfate biology genes involved in early brain development that provide information for future research of the physiological roles and regulation of sulfate homeostasis in human brain.
